# Rheological Behavior of Passion Fruit (*Passiflora edulis*) Peel Extract

**DOI:** 10.3390/gels8090566

**Published:** 2022-09-07

**Authors:** Carlos Arrieta-Durango, Luis Henao-Rivas, Ricardo Andrade-Pizarro

**Affiliations:** Department of Food Engineering, University of Córdoba, Montería 14071, Colombia

**Keywords:** rheology, pseudoplastic, apparent viscosity, elastic behavior

## Abstract

In this work, the rheological behavior of passion fruit peel extract was determined at different temperatures (5–40 °C) and peel content in the extract (40–55% *w*/*w*). The extract was obtained after blanching the passion fruit peels at 95 °C for 5 min, then they were crushed to reduce their size, water was added, and finally, they were subjected to liquefaction and subsequent filtration. Rheological measurements were made using a rheometer with a plate and plate geometry. Extract samples were adequately described by the power-law model exhibiting pseudoplastic behavior, without the presence of thixotropy. The temperature did not influence the flow behavior index, but the consistency coefficient did. The dynamic study (the temperature sweep test) showed that passion fruit peel extract exhibits a more elastic than viscous behavior, typical of a gel.

## 1. Introduction

Passion fruit is the popular name for many different species from genus *Passiflora*, a very common fruit in the tropical and subtropical regions of the globe [1]. The fruit is characterized by its intense flavor and high acidity, which is why it is used as a base for preparing industrialized beverages [2]. The world’s largest producers are Brazil, Ecuador, and Colombia with 60, 12, and 11%, respectively; however, Ecuador is the largest exporter [2,3].

The increase in global demand for passion fruit juice concentrate is mainly due to the growing popularity of multi-fruit juices, exotic-flavored drinks, and multi-vitamin drinks, particularly in Europe and the United States. Processes in the food industry result in many by-products, most of which are organic, and increase the level of environmental pollution. The use of passion fruit pulp leaves behind large quantities of peels because the juice in the fruit represents 30–40%, while the peel represents 50–60%, and the seeds 10–15% [4,5,6].

Passion fruit peel (mesocarp and epicarp) contains a large number of bioactive compounds and polysaccharides, such as pectin and fiber, and can be used as an ingredient in functional foods or to increase viscosity [5,7]. The physicochemical properties of fiber confer viscosity and are comparable to food additives acting as thickening or gelling agents and emulsion stabilizers [5,8,9].

Knowledge of rheological properties is essential for the development of new products, control, and the optimization of the process variables, design, and evaluation of equipment such as pumps, pipes, heat exchangers, mixers, among others, food quality control, and consumer acceptability of a product [10,11].

The rheological parameters of the fluids are calculated from mathematical models proposed for the various operations that form a given process and are generally determined experimentally [10]. The most commonly used models to characterize food products include the Ostwald–De Waele or the power-law model (Equation (1)), Herschel–Bulkley (Equation (2)), Casson (Equation (3)), and Bingham (Equation (4)).

(1) $$\sigma =k\gamma^{n}$$

(2) $$\sigma =\sigma_{0}+k$$

(3) $$\sigma^{0.5}=\sigma_{0}^{0.5}+k\;\gamma^{0.5}$$

(4) $$\sigma =\sigma_{0}+k\;\gamma$$

where: *σ*: shear stress (Pa), *γ*: shear rate (s^−1^), *k*: consistency coefficient (Pa s^n^), *n*: flow behavior index (dimensionless), and *σ*_0_: yield stress (Pa).

The rheological behavior of food fluids is complex and is influenced by numerous factors, such as the shear rate, temperature, solids concentration, moisture content, thermal history, and shear stress [12]. This property has been studied in different food products. For example, rheological data on tuna (*Opuntia ficus Indica*) pulp, pitaya (*Hylocereus undatus*) peel, and tuna (*Opuntia* spp.) peel have been properly fitted to the Ostwald–De Waele and Herschel–Bulkley models, classifying them as pseudoplastic fluids [13,14]. Besides, a decrease in viscosity with an increasing temperature has been reported for borojó pulp [11,15], carrot juice [16], and gabiroba pulp [17]. The temperature dependency on the consistency coefficient can be described by the Arrhenius model (Equation (5)).

(5) $$k=k_{0}\;e^{\frac{Ea}{RT}}$$

where *k*_0_ is the proportionality constant (Pa s^n^), *Ea* is the activation energy (J mol^−1^), *R* is the universal gas constant (J mol^−1^ K^−1^), and *T* is the absolute temperature (K).

This work aimed to characterize, rheologically, the extract obtained from the passion fruit (*Passiflora edulis*), to find alternative applications in the food sector.

## 2. Results and Discussion

### 2.1. The Behavior to the Flow of Passion Fruit Peel Extract

**Figure 1:**
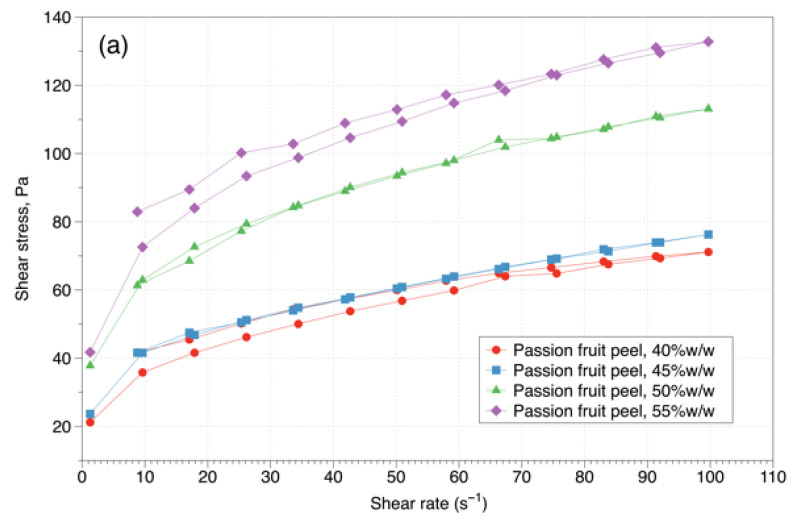

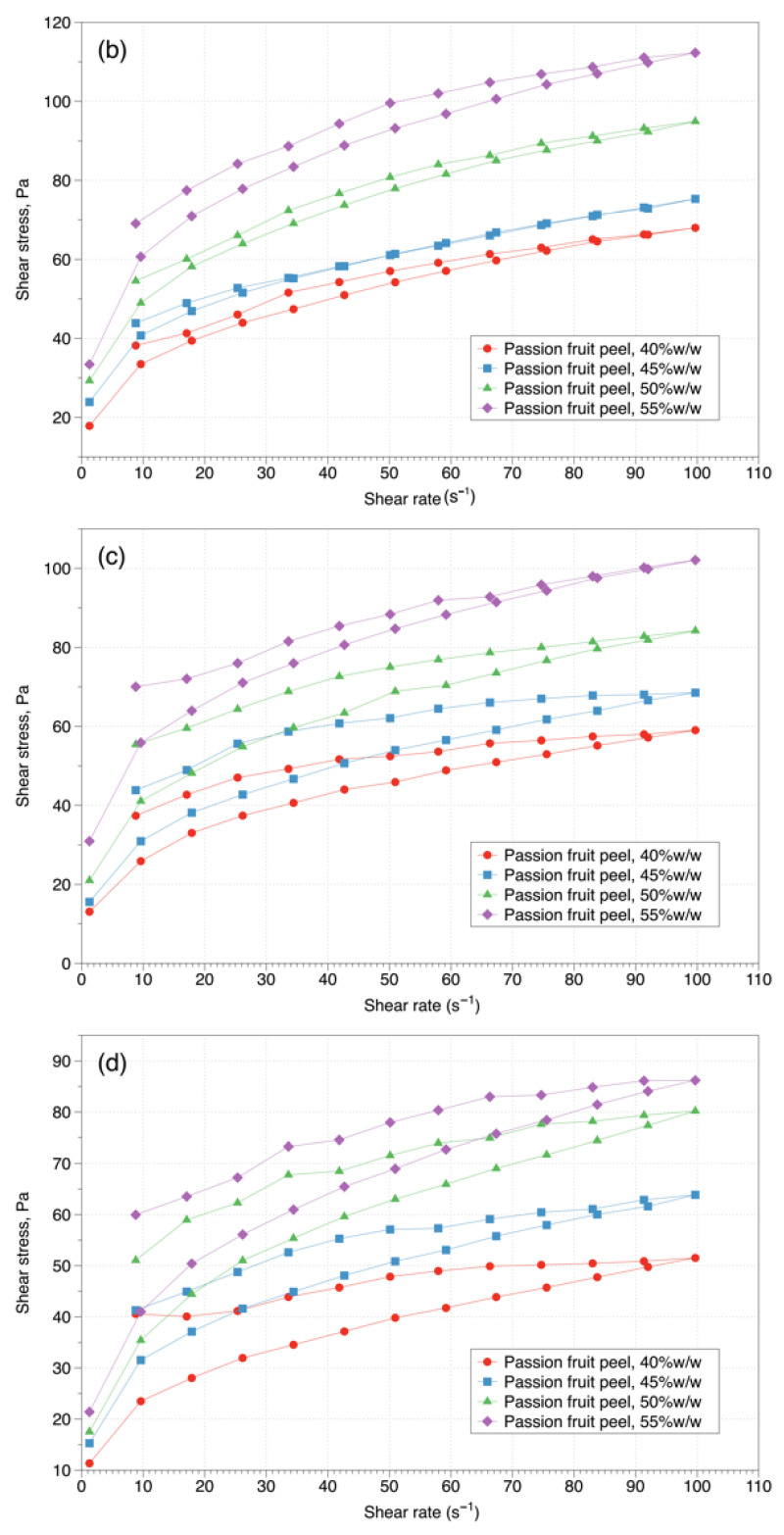
Rheograms of the passion fruit peel extracts at different temperatures. (**a**) 5 °C, (**b**) 10 °C, (**c**) 25 °C, and (**d**) 40 °C.

Figure 1 shows the rheograms of the passion fruit peel extracts at different temperatures, where the ascent and descent curves coincide, showing that all the samples behave independently of time. This behavior may be because there was no rupture of the fluid in the experiment. Therefore, there is no significant variation in viscosity with the time of the application, which coincides with what is reported in sapodilla pulp studied in an interval of 10 to 70 °C [18], and in pasteurized carrot juice evaluated in a range of temperatures from 8 to 85 °C [16].

Passion fruit peel extracts presented a characteristic shear-thinning non-Newtonian behavior, in which the viscosity decreases with increased shear rates (Figure 1). This behavior resulted from the hydrodynamic forces that generated a break in structural units in the extract during shear. The same behavior has been reported for mango peel pectin [19], grapefruit peel [20], passion fruit peel [21], and gabiroba pulp [17,22]. However, for some concentrations (50 and 55% *w*/*w*) the trend lines do not start from the origin. This behavior has been reported for soursop pulp (*Annona muricata L*.) with a concentration of 28°Brix and a temperature range of 30 to 60 °C [23], and pumpkin puree [24].

The rheological models that best represented the rheological behavior of passion fruit peel extracts were the power-law, Herschel–Bulkley, and Casson, presenting values of the coefficient of determination (R^2^) in a range of 0.990 and 0.999 (Table 1). However, the power-law model was selected since the other models, despite presenting high values of R^2^, showed some negative values for the yield stress, *σ*_0_, which is physically not possible, since this stress is the minimum that must be applied to the fluid to overcome the resistance that the material to flow opposes. It should be noted that the power-law model is the most used to represent rheological data of food products. This model has been applied in the rheological characterization of several fruit and vegetable purees [10], guava pulp [25], sapote pulp [26], mango pulp [27], pitaya peel [14], and passion fruit peel pectin [28].

The flow behavior index (n) of all the samples evaluated at the different temperatures was less than one (0.222 to 0.287), which corroborates that the passion fruit peel extract shows a pseudoplastic behavior. Such behavior can be explained by a structural change in the pulp when the shear rate increases, namely the alignment of the biopolymers with the increasing shear rate. Similar values in the flow rate index have been reported for pitaya peel solutions (n = 0.20) [14], solutions of citrus fibre (0.35–0.43) [29], and passion fruit peel pectin [30].

The consistency coefficient (k) presented values in a range from 17.51 to 39.1 Pa s^n^; these values show the high viscosity of the extract if it is compared with the reported for a solution at 1% *w*/*w* of pectin extracted from passion fruit peel (0.21–2.15 Pa s^n^) [28] and fiber solutions obtained from orange by-products (1.52–4.77 Pa s^n^) [29]. However, they were lower than those reported for pitaya peel solutions (161.31 Pa s^n^) [14].

The analysis of variance (ANOVA) shows that none of the factors evaluated (peel count in the extract and temperature) significantly affected the flow behavior index (n). These results are similar to those reported for borojó pulp with a temperature in the range of 0 to 60 °C, where this parameter remained practically constant [15]. However, the consistency coefficient (k) was affected by the peel content in the extract (*p* = 0.0001) and the temperature (*p* = 0.0002). The consistency coefficient increases as the peel content in the extract increase. The increase in k with the peel content in the extract is due to the increased solids content. Similar results were reported for aqueous solutions of gum arabic [30] and jams with added banana peel pectin [31]. This behavior may be due to the agglomerate theory, where the solid phase forms aggregates or nets that trap the dispersing phase [32].

The consistency coefficient decreases with increasing temperature for each percentage of peel in the extract, as follows: 28.45% (40% *w*/*w*), 27.34% (45% *w*/*w*), 23.04% (50% *w*/*w*), and 19.87% (55% *w*/*w*). Similar behavior has been reported for mango pulp [27], and pumpkin puree [24]. This decrease can be explained by the structural breakdown of the extract due to the hydrodynamic forces generated and the increased alignment of its constituent molecules, such as sugars and pectins [23]. The temperature dependence of the consistency coefficient was evaluated by using the Arrhenius model. The values of proportionality constant (k_0_) and activation energy (Ea) are presented in Table 2. The results showed that the Ea values decrease with the increase in peel content in the extract. This indicates that high contents of passion fruit peel in the extract have a greater stabilizing effect, which has been reported by several authors [33,34].

### 2.2. Viscoelasticity of Passion Fruit Peel Extracts

**Figure 2:**
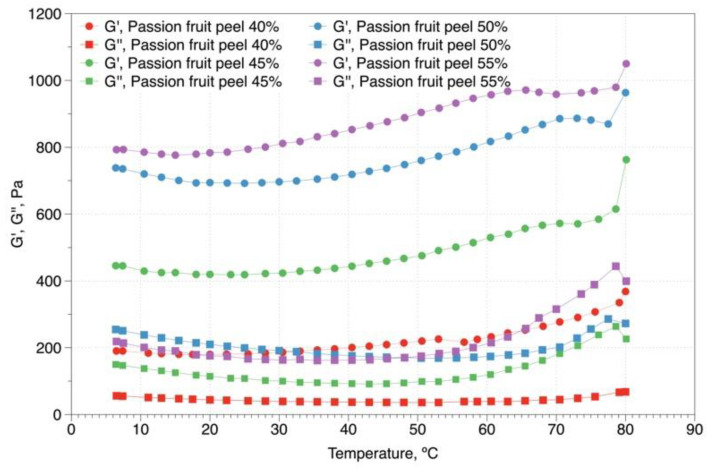
Temperature dependences of storage modulus (G′) and loss modulus (G″) for the passion fruit peel extract.

Figure 2 shows the viscoelastic behavior of passion fruit peel extracts. For all extracts in the evaluated temperature range (5–80 °C), the storage modulus (G′) was higher than the loss modulus (G″), i.e., in passion fruit peel extracts the elastic character predominates over the viscous one, which denotes the thermostability of the fluid. This is a gel-like rheological behavior, where the solid character is attributed to the formation of polymer networks and the liquid or viscous character is due to the presence of the solvent flowing limitedly within the network. This trend has been observed on high methoxyl pectin obtained from tamarillo pulp with the addition of 2 and 3% sucrose [35] and gabioba pulp [22].

On the other hand, the magnitudes of the storage modulus (G′) show an increase with the increase in the percentage of peel in the extract, which indicates a tendency to a more predominant behavior of semi-solid fluid. This higher value in the storage modulus can be attributed to the high pectin content present in the passion fruit peel [6], which implies the formation of a stronger gel, as well as the formation of a more complex structure of long-chain molecules and strongly soluble particles forming a more compact network [36].

## 3. Conclusions

The behavior of passion fruit peel extract in the temperature range 5 to 40 °C and concentrations from 40 to 55% *w*/*w* is represented by the power-law, presenting a pseudoplastic behavior due to the decrease in the apparent viscosity as the shear gradient increases, taking values for the flow behavior index from 0.222 to 0.287.

The temperature does not exert a statistically significant influence on the flow behavior index, while it does exert an influence on the consistency coefficient. On the other hand, the peel content in the extract does not influence the flow behavior index, while the consistency coefficient increases with the increase in the amount of passion fruit peel in the extract.

The viscoelastic behavior of passion fruit peel extracts showed that the storage modulus (G′) was higher than the loss modulus (G″) in all the extracts and temperature range studied, i.e., the samples have an elastic character that predominated over their viscous character, a natural characteristic of a gel. The thermal stability of passion fruit peel extract indicates that it can be used for different applications involving heating, such as for jam and fruit filling in bakery products.

## 4. Materials and Methods

### 4.1. Raw Material

Passion fruits (*Passiflora edulis*) were used, establishing their ripeness by visual identification through the ripening scale of this fruit. The samples were processed in the laboratory of Applied Engineering of the University of Córdoba (Berasteguí, Colombia).

### 4.2. Preparation of Passion Fruit Peel Extract

The passion fruit peels were washed with chlorinated water at 50 ppm and then scalded at 95 °C for 5 min to soften the epicarp and pericarp; the peels were crushed to reduce the size, then water was added according to the established percentage of peel (40, 45, 50, and 55% *w*/*w*), subjected to the homogenization and filtering process.

### 4.3. The Behavior to the Flow of Passion Fruit Peel Extract

Rheological measurements were made using an AR-2000 rheometer (TA Instruments, Chichester, UK), with a plate and plate geometry (40 mm diameter). Passion fruit peel extracts were subjected to a shear rate of 1 to 100 s^−1^, followed by constant shear at 100 s^−1^ for 30 s and finally a decrease in shear rate from 100 to 1 s^−1^ in 2 min [37]. Rheological measurements were performed at different temperatures (5, 10, 25, and 40 °C).

The rheological data were adjusted to the models of Ostwald–De Waele (power-law), Herschel–Bulkley, Casson, and Bingham. The best model was selected considering the coefficient of determination (R^2^) and the root means square error (RMSE).

### 4.4. Temperature Sweep Test for Passion Fruit Peel Extract

The dynamic method was used to study the viscoelastic properties of passion fruit peel extract; tests were carried out within the range of linear viscoelasticity with an AR-2000 rheometer (TA Instruments, Chichester, UK), with a plate and plate geometry (40 mm diameter). During the temperature sweep test, passion fruit peel extracts were heated (5 to 80 °C) and subsequently cooled (80 to 5 °C) at a rate of 2 °C/min, frequency of 1 Hz, and stress of 1 Pa. Dynamic rheological parameters: storage modulus (G′) representing elastic properties, and loss modulus (G″) representing viscous characteristics, were determined.

### 4.5. Experimental Design

A completely randomized design with a 4 × 4 factorial arrangement was used, with the factors peel content in the extract (40, 45, 50, and 55% *w*/*w*) and temperature (5, 10, 25, and 40 °C). All rheological measurements were made in triplicate. The results corresponding to flow behavior and viscoelastic properties were analyzed using an analysis of variance (ANOVA) and Tukey’s test (*p* < 0.05) using JMP 9.1 software.

## Figures and Tables

**Table 1:** Oswald–De Waele model rheological parameters (k and n) of passion fruit peel extracts.

Peel Content in the Extract, *%w/w*	T, °C	k, Pa s^n^	n	Thixotropy, %	R^2^
40	5	23.90 ± 2.45	0.261 ± 0.006	2.45 ± 0.05	0.995
	10	24.35 ± 2.62	0.252 ± 0.007	1.03 ± 0.01	0.996
	25	19.14 ± 0.61	0.237 ± 0.009	1.74 ± 0.02	0.996
	40	17.10 ± 2.39	0.230 ± 0.011	0.20 ± 0.01	0.997
45	5	24.10 ± 2.07	0.250 ± 0.031	2.36 ± 0.01	0.996
	10	25.72 ± 2.85	0.277 ± 0.008	1.65 ± 0.02	0.996
	25	20.94 ± 2.86	0.222 ± 0.015	0.84 ± 0.01	0.996
	40	17.51 ± 1.90	0.261 ± 0.003	1.51 ± 0.01	0.996
50	5	29.85 ± 2.49	0.245 ± 0.011	0.04 ± 0.01	0.996
	10	29.47 ± 1.49	0.236 ± 0.016	1.22 ± 0.01	0.996
	25	23.36 ± 1.17	0.287 ± 0.004	0.04 ± 0.01	0.995
	40	22.97 ± 2.93	0.235 ± 0.007	1.72 ± 0.02	0.996
55	5	39.10 ± 3.16	0.228 ± 0.016	2.55 ± 0.03	0.996
	10	38.47 ± 1.53	0.023 ± 0.017	0.65 ± 0.02	0.996
	25	35.60 ± 3.23	0.263 ± 0.017	1.05 ± 0.01	0.998
	40	31.33 ± 2.12	0.240 ± 0.006	0.51 ± 0.01	0.995

**Table 2:** Arrhenius model for consistency coefficient of passion fruit peel extracts.

Peel Content in the Extract, *%w/w*	k_0_, Pa s^n^	Ea, J mol^−1^ K^−1^	R^2^
5	0.87 ± 0.15	7714.8 ± 0.8	0.944
10	1.03 ± 2.62	7423.1 ± 0.7	0.878
25	2.08 ± 0.61	6163.7 ± 0.9	0.901
40	5.55 ± 2.39	4545.0 ± 0.01	0.966

## Data Availability

Not applicable.
